# Quality assessment and relevant clinical impact of randomized controlled trials on chronic prostatitis/chronic pelvic pain syndrome

**DOI:** 10.1186/s12894-022-01078-5

**Published:** 2022-08-08

**Authors:** Ming Zhao, Qing-He Gao, Sheng-Jing Liu, Ying-Jun Deng, Hao Wang, Wen-Xiao Yu, Jun Guo

**Affiliations:** 1 Department of Andrology, Xiyuan Hospital of China Academy of Chinese Medical Sciences, Beijing, 100091 China; 2 Graduate School, Beijing University of Chinese Medicine, Beijing, China

**Keywords:** Chronic prostatitis/chronic pelvic pain syndrome, Randomized controlled trials, Quality assessment

## Abstract

**Objective:**

This study evaluated the quality of randomized controlled trials (RCTs) on chronic prostatitis/chronic pelvic pain syndrome (CP/CPPS).

**Methods:**

We searched PubMed, Web of Science, and Embase for RCTs (original articles) on CP/CPPS published from database establishment to 2021. The RCT quality assessment was performed using the Consolidated Standards of Reporting of Trials (CONSORT) statement and the improved Jadad scale.

**Results:**

In total, 77 RCTs were included. According to the evaluation, 26 (33.77%) papers presented the description of the specific random methods, only 6 (7.79%) papers described the allocation concealment methods, and 26 (33.77%) articles referred to the “blind method”. Of the RCTs, 34 (44.16%) papers recorded the number of patients who withdrew from the study, and 67 (87.01%) papers reported adverse reactions. However, few reports mentioned the sample size calculation, clinical trial registration, or information about the relevant research programs and funding. In addition, 19 (24.68%) reports had Jadad scale scores of ≥ 4 points, and 58 (75.32%) reports had Jadad scale scores of ≤ 3 points.

**Conclusion:**

To date, the quality of RCT reports on CP/CPPS needs to be further improved, and the results of the RCTs should be accepted and utilized cautiously. It is suggested that researchers should follow the CONSORT statement and the improved Jadad scale to standardize the design and implementation of RCTs to improve the quality of RCTs and provide reliable evidence for the treatment of CP/CPPS.

## Introduction

Prostatitis is a common urinary system disease in adult men. In 1995, the US National Institutes of Health (NIH) reclassified prostatitis into types I (acute bacterial prostatitis), II (chronic bacterial prostatitis), III (chronic prostatitis/chronic pelvic pain syndrome [CP/CPPS]), and IV (asymptomatic inflammatory prostatitis [AIP]) [1]. CP/CPPS refers to a group of diseases in which symptoms such as pain or discomfort and abnormal urination appear in the pelvic area for more than 3 months [2, 3]. CP/CPPS accounts for more than 90% of cases of prostatitis, which is divided into inflammatory (NIH-IIIA) and non-inflammatory (NIH-IIIB) subtypes according to the white blood cell count in [4]. CP/CPPS seriously affects the quality of life of patients. Most patients are not satisfied with the treatment effect, and the incidence of CP/CPPS continues to increase [5]. In recent years, CP/CPPS has become a hot issue in urology and andrology research [6].

Randomized controlled trials (RCTs) provide the strongest recommendations in evidence-based medicine, making them the “gold standard” for evaluating the effectiveness and safety of medical interventions [7]. In recent years, many researchers have conducted a large number of CP/CPPS-related RCTs. The quality of the research and reports will directly affect the judgment of the intervention effect. However, evaluations of RCTs on CP/CPPS are lacking. Thus, this study assessed the quality of RCTs on CP/CPPS using the Consolidated Standards of Reporting of Trials (CONSORT) statement [8] and the improved Jadad scale [9] to propose directions for further studies.

## Methods

### Subjects of the analyses

RCTs on CP/CPPS published from the establishment of databases to December 31, 2021 were identified in PubMed, Web of Science, and Embase using the following search terms: “randomized,” “randomization,” and “randomly.” The search terms for CP/CPPS were prostatitis, prostatism, chronic prostatitis, chronic pelvic pain syndrome, abacterial prostatitis, nonbacterial prostatitis, and prostatodynia. Two reviewers independently searched for RCTs, whereas the different results retrieved by the two reviewers were adjusted and fine-tuned by a third reviewer.

### Quality assessment method

Two reviewers analyzed the RCTs using two quality assessment tools, including the CONSORT statement and the improved Jadad scale. If the data evaluated by the two reviewers were inconsistent, adjustment was performed by a third reviewer.

#### CONSORT statement

The CONSORT statement is a clinical trial report specification formulated by the CONSORT working group. It provides authors with a standardized method to prepare trial results reports, prompts authors to complete transparent reports, and helps them to critically evaluate and explain their results. The CONSORT statement includes a 25-item checklist and a flowchart. The list includes six specifications: title and abstract, introduction (background and objectives), methods (trial design, participants, interventions, outcomes, sample size, randomisation: sequence generation, allocation concealment mechanism, implementation, blinding, and statistical methods), results (participant flow, recruitment, baseline data, numbers analysed, outcomes and estimation, ancillary analyses, and harms), discussion (limitations, generalisability, and interpretation), and other information (registration, trial protocol, and funding). The checklist focuses on how the reported experiment is designed, analyzed, and interpreted. The flowchart presents the progress of all participants in the experiment. The CONSORT statement applies to randomized parallel controlled trials, and strict compliance with the requirements of the items in the list will help authors write clear, complete, and transparent clinical trial reports. If there are deficiencies in the research, then they will be exposed by the transparent reports. Therefore, if an improperly designed experiment is implemented and a transparent report is required, researchers cannot pass the review process for publication of the paper without disclosing the deficiencies of the experiment. The CONSORT statement not only helps researchers to improve the level of clinical research design but also helps editors and peer reviewers to review expert manuscripts and readers rigorously evaluate published literature [10].

#### Jadad scale

The Jadad scale (also known as the Jadad score or Oxford quality scoring system) is a tool for independently evaluating the quality of clinical trial methodology [11]. The improved Jadad scale has a maximum score of seven points, with 1–3 points indicating low quality and 4–7 points indicating high quality. For random sequence generation, when using computer-generated random numbers or similar methods, two points each are awarded for each respective category. When a randomized trial is used but the method of random allocation is not described, one point is awarded for each respective category. When alternate allocation methods are used, such as odd and even numbers, zero points are awarded. For randomization concealment, two points are given when appropriate methods are used to prevent clinicians and subjects from predicting the allocation sequence, such as central or pharmacy control of the allocation plan or the use of containers with consistent serial numbers, on-site computer control, sealed opaque envelopes, or other methods. One point is given when the method of concealment is not clear, such as indicating the use of a random number table or another random allocation plan. Zero points are given when the method of concealment is inappropriate or not used. For blinding, two points are given when the blinding method is appropriate, such as using a completely consistent placebo tablet or a similar method. One point is given when the method is not clear; for example, the test is stated as a blind method, but the method is not described. Zero points are given when the method is not appropriate; for example, it is not appropriate to use double-blind or blind methods, such as comparison of tablets and injections. For withdrawal, one point is given when the number of withdrawals and the reason for withdrawal are described; otherwise, zero points are given.

## Results

### Document retrieval

**Fig. 1:**
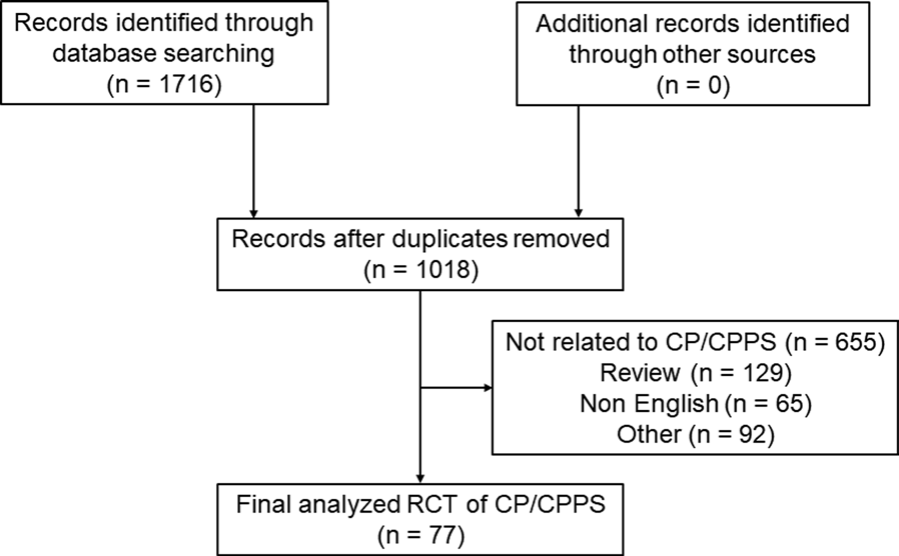
Flow chart of literature screening for RCTs of CP/CPPS. The number of final analysis RCTs of CP/CPPS, included and excluded articles, from the PubMed, Web of Science, and Embase databases

In total, 1716 articles were obtained from the initial search. Of these, 1018 articles were obtained after the first screening, and 77 RCTs were finally included after the second screening. The specific literature retrieval process and results are presented in Fig. 1.

### Basic characteristics of the included studies

The sample of these studies ranged from 17 to 324, with a median of 64, and only 12 (15.58%) of the studies described the process and method of sample size estimation. 21 (27.27%) were multicenter studies, with 2 to 34 research centers. The baseline information was consistent and comparable across studies. The RCTs all aimed to evaluate the effectiveness and safety of the interventions. Regarding the diagnostic criteria for CP/CPPS, 8 (10.39%) studies used the Chronic Prostatitis Collaborative Research Network sponsored by the National Institutes of Health; 4 (5.19%) studies used the European Association of Urology guidelines; 53 (68.83%) studies stated that the diagnosis was made based on history, physical examination, and laboratory tests; and 12 (15.59%) studies only mentioned patients with CP/CPPS, without specifying which diagnostic criteria were used. Of the interventions, 9 (11.69%) studies were alpha1 receptor blockers, including terazosine, doxazosin, and alfuzosin. 5 (6.49%) studies were pollen extracts, and other therapeutic agents included finasteride, palmetto, tamsulosin, silodosin, apremilast, tanezumab, duloxetine, pregabalin, rofecoxib, and zafirlukast, among others. 8 (10.39%) of the studies were extracorporeal shock wave therapy, and other physical rehabilitation treatments included transurethral needle ablation, transurethral microwave thermotherapy with urethral cooling, sono-electro-magnetic therapy, transcutaneous electrical nerve stimulation, repetitive transcranial magnetic stimulation, posterior tibial nerve stimulation, myofascial physical therapy, and cryotherapy, etc. Among the control groups, 44 (57.14%) studies used a placebo or sham treatment, and 33 (42.86%) studies used positive treatment.

### Literature quality evaluation results based on the CONSORT statement

The evaluation of the quality of the literature in the CONSORT statement included the title and abstract, introduction, methods, results, conclusions, and other information. The number and percentage of articles for each topic are presented in Table 1.

**Table 1:** Assessment of the quality of RCTs of CP/CPPS based on the CONSORT statement

Section/Topic	Item No	Checklist item	n	%
*Title and abstract*				
	1a	Identification as a randomised trial in the title	42	54.55
	1b	Structured summary of trial design, methods, results, and conclusions (for specific guidance see CONSORT for abstracts)	67	87.01
*Introduction*				
Background and objectives	2a	Scientific background and explanation of rationale	76	98.70
	2b	Specific objectives or hypotheses	71	92.21
*Methods*				
Trial design	3a	Description of trial design (such as parallel, factorial) including allocation ratio	22	28.57
	3b	Important changes to methods after trial commencement (such as eligibility criteria), with reasons	0	0
Participants	4a	Eligibility criteria for participants	77	100
	4b	Settings and locations where the data were collected	66	85.71
Interventions	5	The interventions for each group with sufficient details to allow replication, including how and when they were actually administered	75	97.40
Outcomes	6a	Completely defined pre-specified primary and secondary outcome measures, including how and when they were assessed	60	77.92
	6b	Any changes to trial outcomes after the trial commenced, with reasons	0	0
Sample size	7a	How sample size was determined	12	15.58
	7b	When applicable, explanation of any interim analyses and stopping guidelines	0	0
Randomisation:				
Sequence generation	8a	Method used to generate the random allocation sequence	26	33.77
	8b	Type of randomisation; details of any restriction (such as blocking and block size)	0	0
Allocation concealment mechanism	9	Mechanism used to implement the random allocation sequence (such as sequentially numbered containers), describing any steps taken to conceal the sequence until interventions were assigned	6	7.79
Implementation	10	Who generated the random allocation sequence, who enrolled participants, and who assigned participants to interventions	8	10.39
Blinding	11a	If done, who was blinded after assignment to interventions (for example, participants, care providers, those assessing outcomes) and how	26	33.77
	11b	If relevant, description of the similarity of interventions	25	32.47
Statistical methods	12a	Statistical methods used to compare groups for primary and secondary outcomes	77	100
	12b	Methods for additional analyses, such as subgroup analyses and adjusted analyses	15	19.48
*Results*				
Participant flow (a diagram is strongly recommended)	13a	For each group, the numbers of participants who were randomly assigned, received intended treatment, and were analysed for the primary outcome	36	46.75
	13b	For each group, losses and exclusions after randomisation, together with reasons	34	44.16
Recruitment	14a	Dates defining the periods of recruitment and follow-up	18	23.38
	14b	Why the trial ended or was stopped	1	1.30
Baseline data	15	A table showing baseline demographic and clinical characteristics for each group	57	74.03
Numbers analysed	16	For each group, number of participants (denominator) included in each analysis and whether the analysis was by original assigned groups	57	74.03
Outcomes and estimation	17a	For each primary and secondary outcome, results for each group, and the estimated effect size and its precision (such as 95% confidence interval)	49	63.64
	17b	For binary outcomes, presentation of both absolute and relative effect sizes is recommended	19	24.68
Ancillary analyses	18	Results of any other analyses performed, including subgroup analyses and adjusted analyses, distinguishing pre-specified from exploratory	7	9.09
Harms	19	All important harms or unintended effects in each group (for specific guidance see CONSORT for harms)	67	87.01
*Discussion*				
Limitations	20	Trial limitations, addressing sources of potential bias, imprecision, and, if relevant, multiplicity of analyses	57	74.03
Generalisability	21	Generalisability (external validity, applicability) of the trial findings	61	79.22
Interpretation	22	Interpretation consistent with results, balancing benefits and harms, and considering other relevant evidence	57	74.03
*Other information*				
Registration	23	Registration number and name of trial registry	16	20.78
Protocol	24	Where the full trial protocol can be accessed, if available	3	3.90
Funding	25	Sources of funding and other support (such as supply of drugs), role of funders	6	7.79

#### Title and abstract

Among the included 77 RCTs, 67 (87.01%) articles mentioned “randomized” and “controlled” in the title and abstract, and the other 10 (12.99%) articles did not specify the type of study in the title and abstract.

#### Randomization, blinding, and allocation hiding

Although the 77 included RCTs mentioned “randomized,” only 26 (33.77%) papers mentioned the specific randomization methods. Among them, 17 (33.77%) papers generated random assignment sequences by computer, and 9 (11.69%) papers used the order of patient visits as the grouping method. Only 26 (33.77%) articles mentioned the “blind method,” but they did not explain how the method was implemented. 6 (7.79%) studies mentioned allocation hiding.

#### Subjects and test procedures

36 (46.75%) articles mentioned the recruitment of subjects and the baseline situation of subjects. Conversely, only 6 (7.79%) articles used graphs to present the baseline data. Only 34 articles (44.16%) recorded the number of patients who withdrew from each group after random grouping. 16 (20.78%) articles used flowcharts to record the test process.

#### Adverse reactions

Of the 77 included RCTs, 67 (87.01%) studies reported whether adverse events occurred during the study, and none of the remaining literature stated whether adverse events occurred before or after the trial.

#### Statistical methods

Among the 77 included RCTs, all articles specifically described the statistical methods used to compare the primary and secondary outcome indicators of each group, and 15 (19.48%) articles described the methods of additional analysis.

#### Clinical registration, plan announcement, and funding description

Among the included studies, only 16 (20.78%) documents stated that they were registered on various registration platforms, only 3 (3.90%) mentioned the relevant trial schemes and 6 (7.79%) mentioned funding status.

#### Interpretation of results

Among the 77 included RCTs, 57 (74.03%) studies described the limitations and reasons for the trial in the discussion section. And 57 (74.03%) studies provided explanations that are consistent with the experimental results. Meanwhile, 61 (79.22%) articles provided negative results.

### Literature quality evaluation results based on the improved Jadad scale

Among all 77 articles evaluated by the improved Jadad scale, only 19 (24.68%) had a score of ≥ 4. Meanwhile, 58 (75.32%) were low-quality studies (score ≤ 3), and little of them mentioned random hiding and execution. The number and percentage of articles for each score are presented in Table 2.

**Table 2:** Assessment of the quality of RCTs in the treatment of CP/CPPS based on improved Jadad scale scores

Improved Jadad scale score	Number of articles (n)	Percentage (%)
0	4	5.19
1	10	12.99
2	5	6.50
3	39	50.65
4	10	12.99
5	4	5.19
6	4	5.19
7	1	1.30

## Discussion

According to the CONSORT statement and the improved Jadad scale, this study evaluated the literature on RCTs in the treatment of CP/CPPS. The results illustrated that the quality of the studies in this field needs to be improved in several aspects.

As we all know, irregular presentation of the title and abstract may influence the reader’s judgment of the literature and affect whether the literature can be identified by the searcher. Therefore, we recommend that the authors clearly label the title and abstract with “randomized” and “controlled” to indicate that the study was an RCT. However, this does not mean that the authors did not use randomization in their study. Appropriate sample size estimation is an important part of clinical trials [12]. The sample size must be sufficient to reliably answer the relevant questions raised by the research hypothesis and meet the accuracy and reliability of statistics; at the same time, the sample size should not be excessive to avoid waste. Estimation of the sample size is the basis for the credibility of the research results. Therefore, future clinical studies of CP/CPPS should pay attention to the estimation of sample size.

Although most studies did not explicitly state the method of randomization, this does not mean that these studies did not use randomization or that randomization occurred in error. Therefore, we further used the improved Jadad scale to better assess randomization, blinding, and allocation concealment. Whether the random sequence is reasonable directly affects the feasibility and credibility of clinical RCTs. Blinding is one of the important measures for controlling bias in RCTs. Implementation of a blinding method can effectively control the bias caused by human-made differences between groups [13]. Adopting the correct method design (such as the random method, blind method, and allocation concealment) is an important measure for reducing the bias of clinical research. An incorrect design may exaggerate research results.

A flow chart can intuitively record the collection of cases, the status of the test, and the number of withdrawals, but most researchers do not pay sufficient attention to the test process. Adverse event reporting is an important part of RCTs. Data on adverse reactions help readers to judge the safety of various therapies, and they can have a major impact on judging the acceptability and practicality of interventions. In addition, our results suggested that researchers do not pay enough attention to data statistics. Statistics, as the foundation of scientific research, is an important tool for medical scientific research [14]. The correct application of statistical methods directly affects the quality of medical scientific research and the level of scientific research papers.

Moreover, clinical registration, plan announcement, and funding description are conducive for readers to judge the transparency and reliability of CP/CPPS clinical research. Our results also illustrate that researchers’ awareness of clinical research registration is relatively weak at this stage. Clinical trial registration is a basic requirement for high-level journals to publish articles. The articles in the clinical registry are only available in the last few years. Future CP/CPPS clinical research should ensure that studies are appropriately registered. Inadequate discussion of study findings may lead to publication bias, thereby exaggerating the effects of interventions. These studies we analyzed are relatively well done in the process of discussion.

The CONSORT statement has a total of 37 entries for the 25-item checklist, and the number of entries met by these studies ranged from 6 to 25. If both the number of entries and the Jadad score were high, we considered these studies to be relatively reliable and their quality to be acceptable. The screening was performed with the condition of ≥ 20 entries and a Jadad score of ≥ 4. A total of 15 studies met the requirements, and we considered these to be more formal and scientifically reliable papers.

Through quality assessment, we found that some lower-quality RCT studies still managed to be published, which may include the following reasons. First, the treatments in these studies may be relatively new and the studies are still in the exploratory phase. Second, these studies were able to present scientific evidence that addressed some clinical questions or contradictions. Finally, although they do not follow a standardized protocol to improve the quality of the study, their data are realistic.

Generally speaking, the results of our study indicated that the quality of RCTs in the treatment of CP/CPPS needs to be improved, and care should be taken when accepting and using the results. These inadequacies included a lack of information on the nature of the experiment in the title, the estimated sample size, the absence of random grouping or unreasonable methods, a lack of blinding, the absence of graphs to represent the baseline data of subjects, no records on patient withdrawal and elimination, a lack of details about adverse events, and the absence of clinical registration. This missing information may cause readers to question the correctness and rationality of the relevant research design, making it difficult to promote the clinical application of treatments for CP/CPPS. When conducting future research, researchers should follow the internationally recognized CONSORT statement and use the improved Jadad scale to standardize the design and implementation of RCTs; correctly select randomization, blinding, and statistical methods; and pay attention to adverse reaction reports and clinical registration instructions. This strategy to improve the quality of RCTs will provide a more reliable basis for the promotion of clinical applications for the treatment of CP/CPPS.

## Data Availability

The datasets used and/or analyzed during the current study are available from the corresponding author on reasonable request.

## Abbreviations

RCT: Randomized controlled trial
CP/CPPS: Chronic prostatitis/chronic pelvic pain syndrome
CONSORT: Consolidated standards of reporting of trials
